# Progress in research on the role of exosomal miRNAs in the diagnosis and treatment of cardiovascular diseases

**DOI:** 10.3389/fgene.2022.929231

**Published:** 2022-10-04

**Authors:** Jinyu Xu, Weitie Wang, Yong Wang, Zhicheng Zhu, Dan Li, Tiance Wang, Kexiang Liu

**Affiliations:** Department of Cardiovascular Surgery, The Second Hospital of Jilin University, Changchun, China

**Keywords:** exosomes, cardiovascular, cardio, miRNA, treatment

## Abstract

Cardiovascular diseases are the most common diseases threatening the health of the elderly, and the incidence and mortality rates associated with cardiovascular diseases remain high and are increasing gradually. Studies on the treatment and prevention of cardiovascular diseases are underway. Currently, several research groups are studying the role of exosomes and biomolecules incorporated by exosomes in the prevention, diagnosis, and treatment of clinical diseases, including cardiovascular diseases. Now, based on the results of published studies, this review discusses the characteristics, separation, extraction, and identification of exosomes, specifically the role of exosomal miRNAs in atherosclerosis, myocardial injury and infarction, heart failure, aortic dissection, myocardial fibrosis, ischemic reperfusion, atrial fibrillation, and other diseases. We believe that the observations noted in this article will aid in the prevention, diagnosis, and treatment of cardiovascular diseases.

## 1 Introduction

Cardiovascular diseases affect the quality of life of the affected patients and are associated with a high mortality rate. Recently, many studies have reported that the incidence and mortality rate associated with cardiovascular diseases is increasing annually and that they are the most common diseases that affect adults, in particular, middle-aged adults. The annual mortality percentage due to cardiovascular diseases has reached 30–40% (Ragusa et al., 2015), which surpasses that caused by cancer, and is expected to increase in the next decade. Therefore, treatment of cardiovascular diseases has always been the focus of clinical research. Recent studies have revealed that exosomes contribute to the physiological and pathological mechanisms of cardiovascular diseases (Wang Y. et al., 2019; Mashouri et al., 2019) by transmitting signals between cells; in particular, the exosomal miRNAs regulated the expression of various signaling pathway members. We have reviewed the role of exosomes and exosomal miRNAs in cardiovascular diseases (Zheng et al., 2021), but there was lack of relevant reports at that time. Therefore, we have reviewed the progress in the research on the role of exosomal miRNAs in the pathogenesis, diagnosis, treatment, and other aspects of cardiovascular diseases in this article.

## 2 Overview of exosomes

**FIGURE 1:**
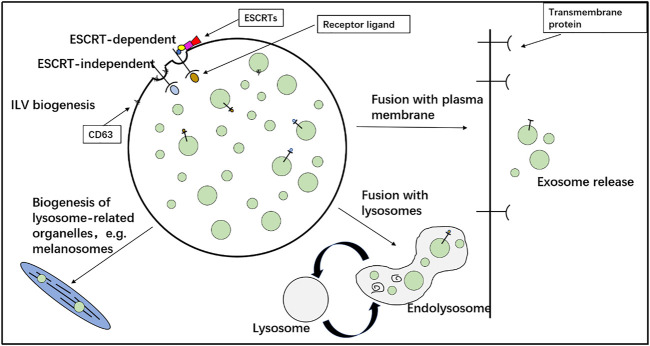
Formation of exosomes. Initially, the cytoplasmic membrane is in the early inner body, which is again formed in the advanced inner body to the inner bud and finally secreted out of the cells.

Exosomes are a subpopulation of cell-secreted extracellular vesicles; the process begins with cell membrane invagination, and exosomes are then secreted by the cell after incorporating active factors such as proteins and nucleic acid fragments. The earliest exosomes were called small extracellular vesicles, which were found by Johnstone et al. (1987) while studying reticulocytes. In 1987, they were renamed as exosomes, and in 2018, the international scientific community uniformly defined the size of the exosomes to be about 30–100 nm. Exosomes are enclosed by a relatively stable lipid bilayer, and they appear as flat cup-shaped balls under the electron microscope (Edgar, 2016; Pathan et al., 2019; Tschuschke et al., 2020). The formation of exosomes (Figure 1) includes three steps: initially, the cytoplasmic membrane is found in the early inner body, which is again formed in the advanced inner body to the inner bud, and finally secreted out of the cells. This process relies on the endosomal sorting complex required for transport (ESCRT). Most types of cells, such as smooth muscle cells, stem cells, lymphocytes, platelets, and fat cells, secrete exosomes. Exosomes contain biomolecules such as nucleic acids (mRNA, miRNA, and DNA), lipids, and proteins (heat shock proteins such as HSP60, transmembrane 4 superfamily (TM4SF), and CD63) depending on the type and state of the cells secreting them. They are extensively distributed in various body fluids, such as blood, cerebrospinal fluid, and pleural effusion, and circulate in the body, participating in the exchange of cytochemical information (Wang X. et al., 2019). The biomolecular cargo in the exosomes changes under different pathological conditions, such as hypoxia and inflammation (Vizoso et al., 2017). The lipid bilayer membrane structure of the exosome is relatively stable, protecting itself and its labile cargo of proteins and RNAs from the body fluid. In summary, exosomes have a wide range of characteristics (Wang X et al., 2018) and play important roles in the exchange of cellular information (Wei et al., 2021). As they reflect the pathophysiological state of source cells, they may be used for the prevention, diagnosis, and even treatment of cardiovascular diseases.

## 3 Separation and extraction of exosomes

**FIGURE 2:**
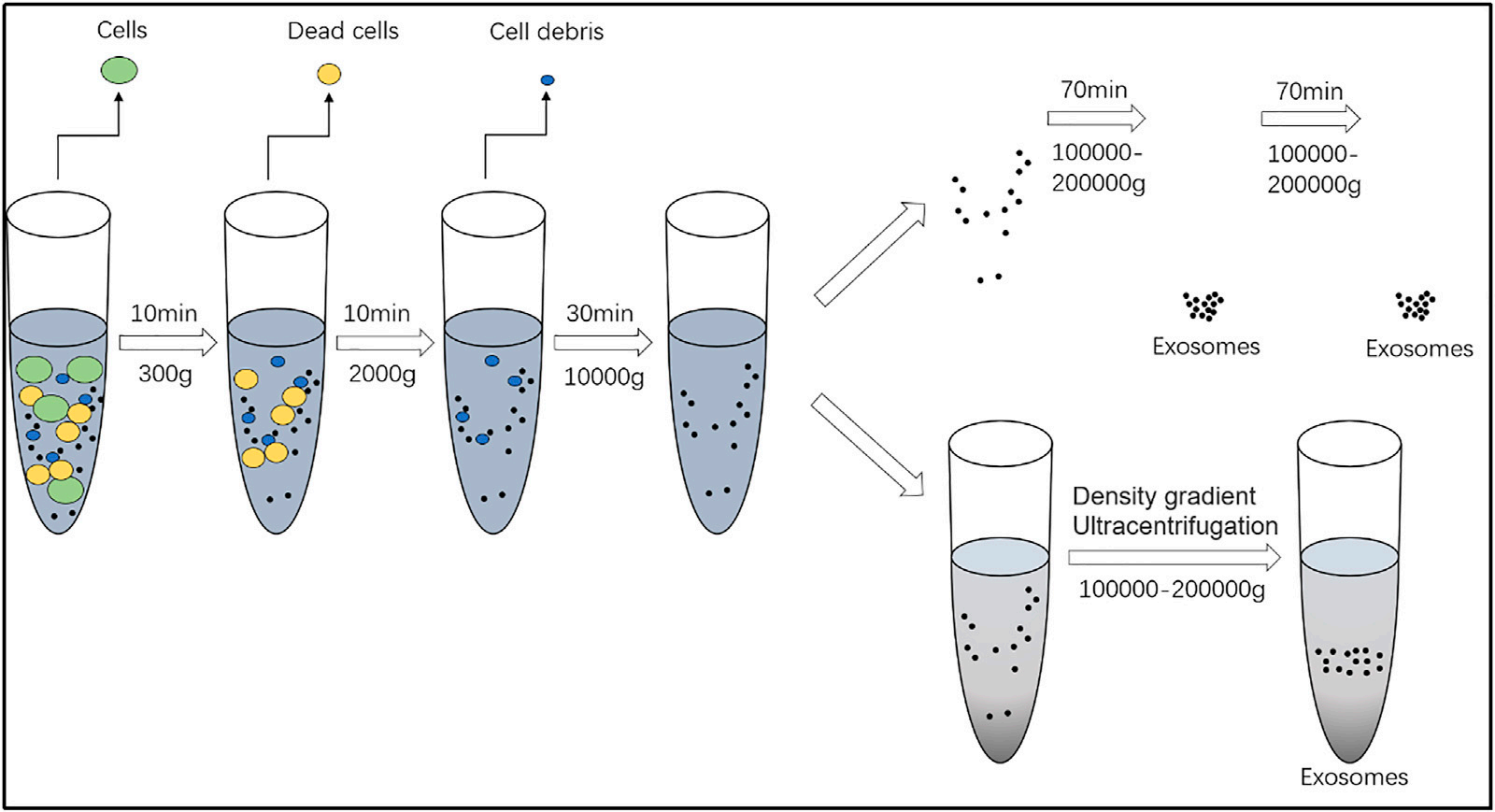
Separation and extraction of exosomes. Centrifugal speeds of 300 × *g*, 2,000 × *g*, and 10,000 × *g* are used to remove cells and debris, while apoptotic bodies and large vesicles are eventually obtained at 100,000 × *g*.

The settling factors in a solution vary with substances, which determine the centrifugal speed at which they can be precipitated. Differential centrifugation is commonly used for obtaining exosomes. Centrifugal speeds of 300 × *g*, 2,000 × *g*, and 10,000 × *g* are used to remove cells and debris, while apoptotic bodies and large vesicles are eventually obtained at 100,000 × *g*; combining this with a 0.22-μm or 0.45-μm aperture filter can increase purity, if necessary, and the pellet obtained can be resuspended in phosphate-buffered saline to obtain pure exosomes (Jeppesen et al., 2014; Momen-Heravi, 2017) (Figure 2). The advantage of this method is that highly pure preparations of lipoprotein particles and proteins can be obtained at a low cost; however, the method is time-consuming. In addition, the structure of the exosomes is destroyed by the centrifugal shear. Purification of exosomes requires appropriate sample viscosity, rotor, and rotation radius (O'Brien et al., 2018). Therefore, density gradient centrifugation is now used for purifying exosomes. The density gradient is gradually increased from the top to the bottom of the centrifuge tube using a common medium such as iodixanol. The specific operation is divided into the equivalent gradient centrifugal method and rate zone centrifugal method based on the density of particles in each of the density gradient zones and the settlement rate of different particles, respectively (Doyle and Wang, 2019).

Polymers such as polyethylene glycol are used to form a mesh structure in the solution, which increases the binding force of the hydrophobic protein and lipid molecules and disengages them from the solution. As lectins of exosomal glycoproteins combine with sugar chains, the dispersibility and solubility of the exosomes may change, and they can be obtained *via* centrifugation at low speed (Ramirez et al., 2018; Wang et al., 2021). This method is simple and time-saving, and the exosomes are less damaged. However, the purity of the exosomes is low; in particular, when the exosomal fluid component is complex, the proteins present in the liquid, such as fibrinogen, and lipoprotein particles, and part of the bubble precipitate together, rendering separation challenging, which may affect the results of the study (Helwa et al., 2017). Therefore, samples are pretreated with protease K to increase the purity of exosomes (Moon et al., 2019). Nonetheless, this method is not preferred for extracting exosomes.

**FIGURE 3:**
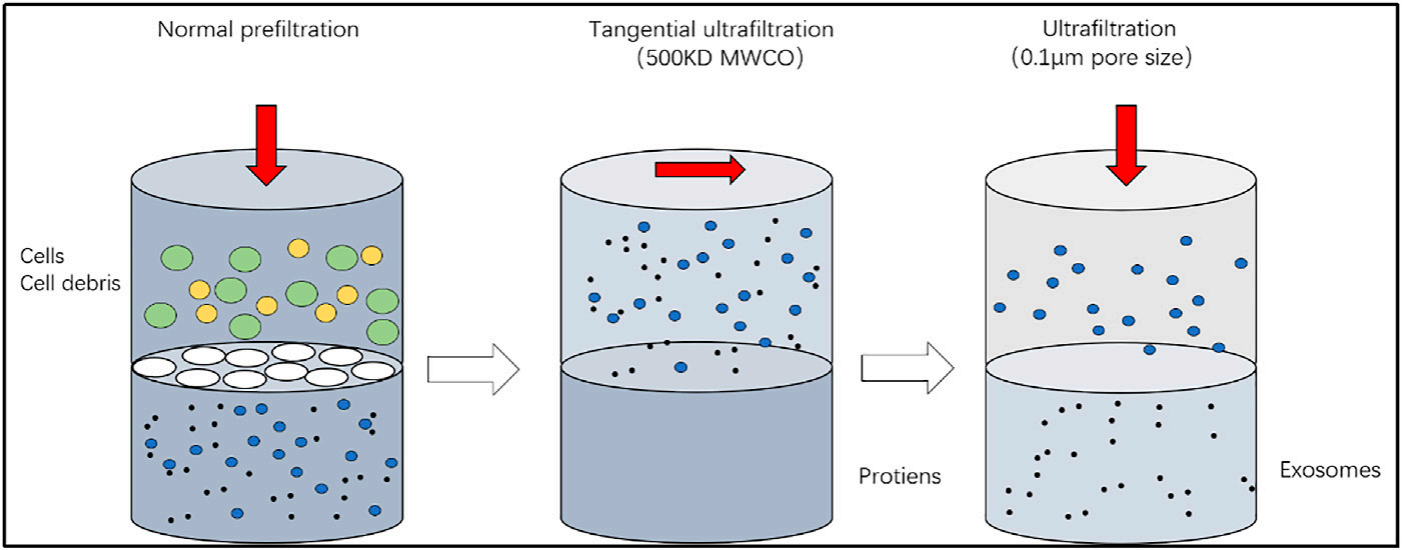
Ultrafiltration. The principle is based on the size of the exosomes, and the sample is separated using a special aperture filter, which removes molecules such as proteins, while retaining the exosomes.

Ultracentrifugation and pressure ultrafiltration are time-saving and efficient methods for extracting exosomes (Figure 3). The principle is based on the size of the exosomes, and the sample is separated using a special aperture filter, which removes molecules such as proteins, while retaining the exosomes (Li et al., 2017). Low purity of the exosomes obtained is also the disadvantage of this method because substances with a diameter similar to that of the exosomes are also intercepted at the same time; in addition, the ultrafiltration efficiency may be affected if the ultrafiltration membrane is blocked or cracked (Ding et al., 2021). Therefore, the non-symmetric flow field separation method is used, in which the force field is applied in different directions, and the filtrate, flowing at different speeds, is formed at an angle with the filter membrane during the flow. This considerably reduces the chances of filter membrane blockage. In addition, a combination of different detection methods can achieve sub-selected sorting of different vesicles (Zhang and Lyden, 2019; Lin et al., 2020; Yang et al., 2020). However, improvements in the amount of time required for the procedure and yield are still required.

Exosomes possess a special membrane protein, which can be used to extract exosomes using an immune-affinity membrane (Li et al., 2017). This method works on the principle of antigen–antibody recognition, in which the specific antigen is attached to the membrane *via* magnetic beads. However, this method is expensive and low-yielding and has hence not been used widely. Exosomes can be extracted using various other methods, such as chromatography, molecular sieve analysis, and the emerging microfluidic technique, each of which has its own characteristics, advantages, and disadvantages. In the clinic, we always use a combination of multiple methods to improve the efficiency and purity of the exosomes.

## 4 Identification of exosomes

After extraction, the exosomes have to be identified for downstream experiments. The identification methods vary depending on the physical and chemical properties of the exosomes (including size, morphology, concentration, and protein markers present).

We have mentioned that exosomes are cup-shaped and 30–100 nm in diameter. This feature can be used in nanoparticle tracking analysis, dynamic light scattering, and adjustable resistance pulse sensing. The exosomes in the sample move according to the principle of particle Brownian movement, as well as their size and of the surrounding medium on the exosomes (Tang et al., 2021). The advantage of this method is that it is time-saving, although the specificity is poor. Thus, proteins and exosomes of similar sizes cannot be distinguished. A transmission electron microscope or scanning electron microscope can also be used to identify the “cup” structure of the exosomes (Jung and Mun, 2018).

The most common method involves identification of specific protein markers harbored by exosomes using nano-fluorescent activated cell sorting (FACS) and Western blotting. In nano-FACS, because of fluorescent antigen–antibody reactions, exosome vesicles linked to beads can be sorted using flow cytometry. Previously, we have mentioned that exosomes harbor HSP60, TM4SF, CD63, CD9, CD81, and other specific protein markers. Thus, they can be identified by detecting the expression of specific proteins using Western blotting (Huang L. H et al., 2021). The disadvantage of this method is that it is time-consuming; however, impurities in the preparation can be avoided, and the exosomes can be identified accurately. At the same time, the concentration of the exosomes can be determined.

## 5 Function and application of exosomes

As mentioned previously, exosomes are formed as a result of cytoplasmic invagination and efflux. Previously, scientists believed that exosomes cleared cellular debris such as biomolecules that are not required by the cells. Currently, exosomes are known to regulate apoptosis and participate in immune response *via* the nuclear factor kappa-B (NF-κB) signaling pathway (Lindenbergh et al., 2018; Lu et al., 2018; Aghabozorgi et al., 2019; Lindenbergh et al., 2019; Li and Wang, 2021; Lindenbergh et al., 2020).

**FIGURE 4:**
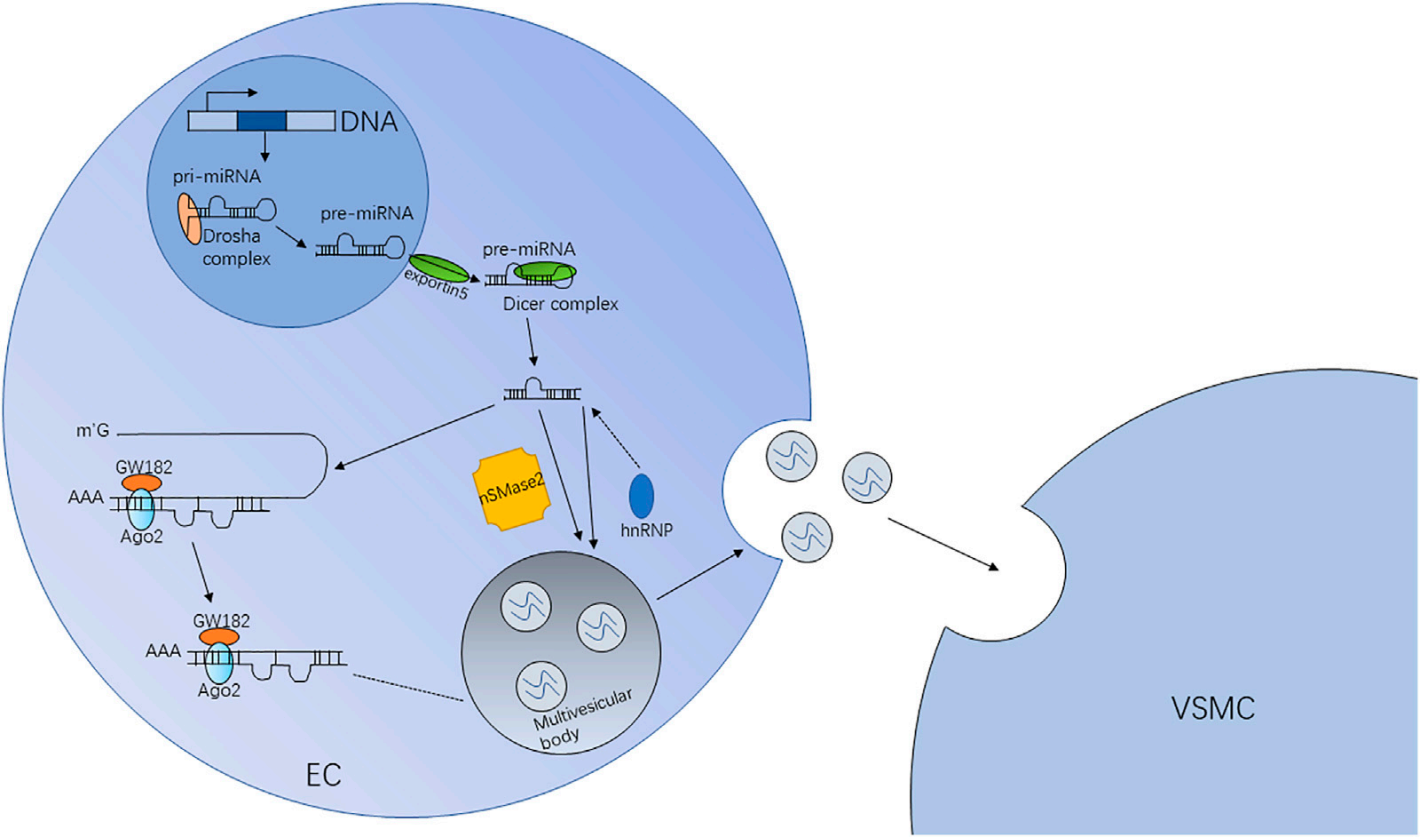
Pathway and mechanism of exosomal miRNAs. In ECs, miRNA genes are transcribed into primary miRNAs (pri-miRNAs) initially and then form precursor miRNAs (pre-miRNAs) processed by the Drosha complex. Because of the exportin5 complex, the pre-miRNAs are exported into the cytoplasm. Finally, through the digestion of the Dicer complex, the pre-miRNAs become mature. Mature miRNAs are sorted into exosomes depending on the nSMase2-dependent pathway, the hnRNP pathway, etc.

Exosomes can also mediate pathological processes, which is why they are being actively researched. Exosomes contribute to the pathogenesis of many diseases. Currently, their role in cancer is being extensively studied. Tumor-derived exosomes carry information such as nucleic acids and proteins and play important roles in the development and metastasis of tumors (Ruivo et al., 2017; Kogure et al., 2020). Exosomes were used clinically based on their functions and characteristics. First, exosomes can protect their cargo (miRNAs and proteins) (Kumar et al., 2020) because of their phospholipid bilayer structure. At the same time, they are widely distributed in the body and have long half-lives (Nam et al., 2020). In addition, exosomes are small; hence, they have strong penetration power (Szabo and Momen-Heravi, 2017) and can freely shuttle between cells and evade phagocytic effects (Figure 4). Second, the specific protein markers incorporated by the exosomes play an important role in the diagnosis of diseases. For example, Taylor and Gercel-Taylor (2008) first proposed that exosomal miRNA-21 can act as a marker of ovarian cancer and even determine the progress of the disease. The therapeutic effects of exosomes vary with their type and concentration (Beltrami et al., 2017; Chuppa et al., 2018; Powers et al., 2020).

As exosomes can regulate apoptosis, they can be used for therapy. The biomolecules (nucleic acid information and proteins) incorporated by the exosomes can be suppressed or promoted for treating diseases. For example, Rong et al. (2016) have shown that the inhibition of T lymphocyte proliferation can be reduced by suppressing the expression of exosomal TGF-β, thereby inhibiting tumor metastasis. In summary, the application prospects of exosomes are broad, and the application of miRNAs in cardiovascular diseases has been discussed subsequently.

## 6 Role of exosomal miRNAs in cardiovascular diseases

**FIGURE 5:**
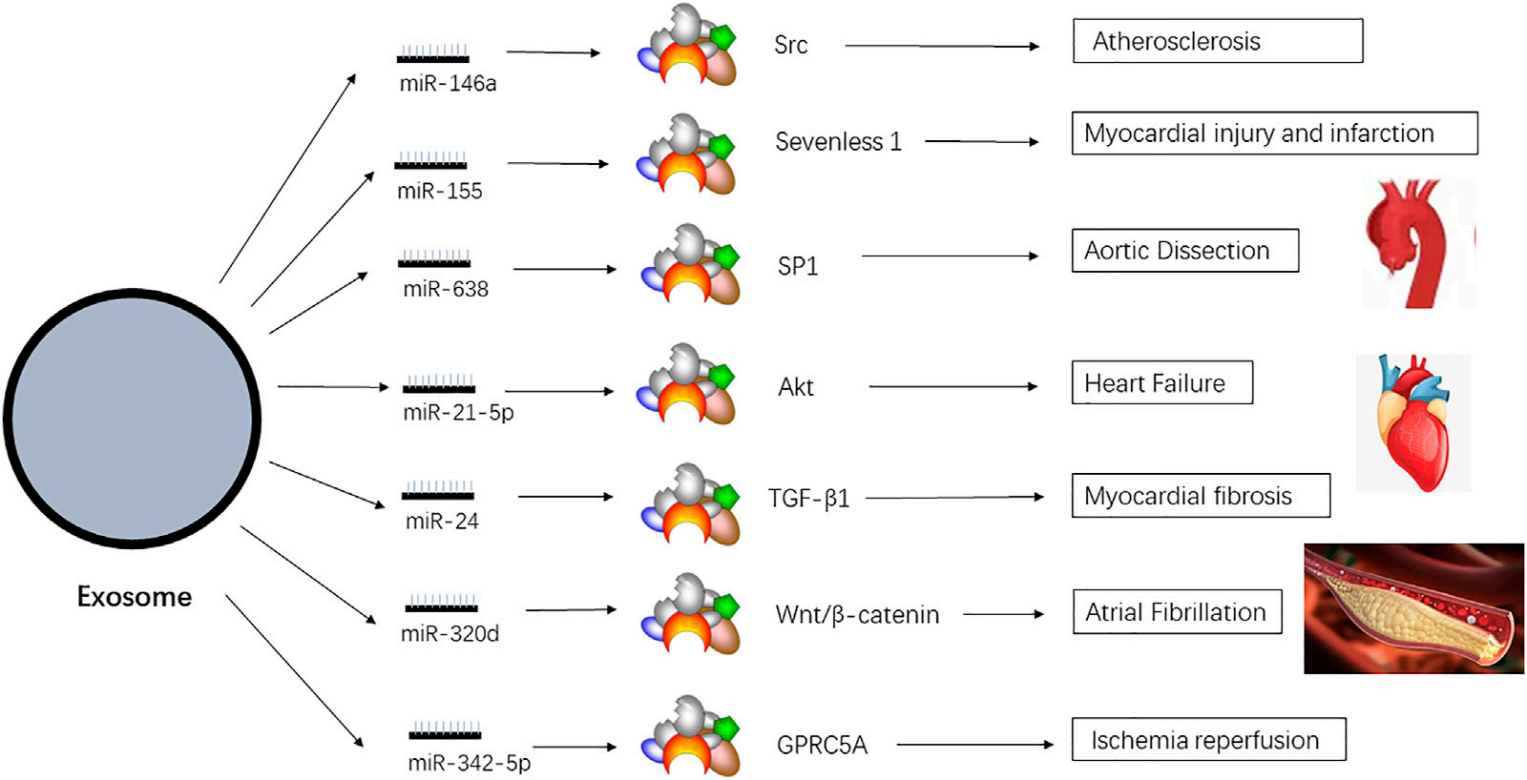
Exosomal miRNAs which are related to cardiovascular diseases. There are various miRNAs in the exosomes, and they depend on the special pathway to affect the diseases.

Since their discovery, various signaling molecules have been found in exosomes. In particular, exosomal miRNAs transmit information between cardiac cells *via* endocytosis and fusion. The exosomal miRNAs participate in transcriptional regulation and affect the occurrence and development of various diseases, especially cardiovascular diseases (Figure 5). The high incidence and mortality associated with cardiovascular diseases have boosted research on their treatment and prognosis. Many studies have reported the involvement of exosomal miRNAs in cardiovascular diseases (Table 1), such as atherosclerosis and heart failure.

**TABLE 1:** Exosomal miRNAs related to cardiovascular diseases.

Cardiovascular diseases	Related exosomal miRNAs	References
Atherosclerosis	miR-146a, miR-223, miR-16, and miR-21	Nguyen et al., 2018; Zhang et al., 2019b; Wang X. et al., 2018
Myocardial injury and infarction	miR-17, miR-324, miR155, miR-1, miR-208a, and miR-192	Sun et al., 2019; Pan et al., 2019; Hu et al., 2019; Han et al., 2020; Wang et al., 2017; Li et al., 2018; Vanni et al., 2017
Heart failure	miR-21, miR-146a, miR-425, miR-744, and miR92b-5p	Cheng et al., 2022; Qiao et al., 2019; Ma et al., 2018; Emanueli et al., 2016; Wu et al.,2018
Aortic dissection	miR-155, hsa-miR-26a-5p, miR-320, miR-146a-5p, miR-134–5p, miR-223–3p, miR-599, hsa-miR-182–5p, and miR-145	Choi et al., 2018; Aschacher et al., 2022; Liao et al., 2018; Ji et al., 2019; Xue et al., 2019; Aschacher et al., 2021; Wang et al., 2019a; Mimler et al., 2019; Wu et al., 2019
Myocardial fibrosis	miR-21–5p, miR-294, miR-24, miR-125b-5p, and miR-146a	Frangogiannis, 2019; Gollmann-Tepeköylü et al., 2020; Moghaddam et al., 2019
Ischemia reperfusion	miR-342–5p, miR-30a, miRNA-181a, miR-148a, miR-150, miR-21, and miR-126	Hou Z. et al., 2019; Chen et al., 2017; Jia et al., 2017; Wen et al., 2018; Zhou H. et al., 2019; He et al., 2020
Atrial fibrillation	miR-1, miR-328, miR-29a-3p, miR-320d, miR-486–5p, miR-107, miR-103a-3p, miR-223–5p, miR-223–3p, miR -3126–5p, and miR-27b-3p	Terentyev et al., 2009; Park et al., 2017; Lu et al., 2010; Zhao et al., 2016; Mun et al., 2019; Wang et al., 2019b; Liu et al., 2020

### 6.1 Role of exosomal miRNAs in atherosclerosis

Atherosclerosis is the most common and important cardiovascular disease. It is a chronic progressive inflammatory reaction with no symptoms in the early stage. With deterioration of symptoms, lipids are deposited inside the blood vessels and the vascular wall stiffens, which reduces vascular compliance, resulting in vascular wall damage (Heo and Kang, 2022). Recent studies have shown that exosomes participate in vascular calcification by enabling information exchange between cells and play an important role in vascular atherosclerosis (Zhang and Huang, 2021). In particular, the miRNAs present in exosomes are one of the main agents that regulate atherosclerosis. The main pathological process of vascular calcification involves an increase in the expression of osteogenesis-related genes (Yang et al., 2019). According to a report, miR-146a in macrophages can promote the calcification of vascular smooth muscles by inducing oxidative stress, promoting the wrap of macrophages up the vascular wall and reducing cell migration (Nguyen et al., 2018; Zhang YG. et al., 2019). The exosomes produced by bone marrow mesenchymal stem cells transfected with miR-146a lowered the expression of the gene encoding thioredoxin-interacting protein, thereby partially inhibiting calcification (Wang Y. et al., 2018). Another study showed that the exosomes harboring miR-223 were released by platelets (Lazar et al., 2021). After entering smooth muscle cells, the exosomes regulated the proliferation and migration of cells, affecting the progress of endothelial inflammation and atherosclerosis. The use of indophenol can reduce the expression of miR-223, limiting the development of atherosclerosis (Shi et al., 2020).

In addition, several reports show that exosomes influence the expression of anti-inflammatory and proinflammatory factors *via* the NF-κB pathway, which can affect atherosclerosis. Lu et al. (2019) found that exosomes transmit inflammatory cytokines and miRNAs to the receptor cells and activate the NF-κB pathway, which can cause endothelial inflammation and atherosclerosis. Shi et al. (2019) found that exosomes containing serum HSP27 and NF-κB were activated by the receptor, promoting the release of IL-10, thereby inhibiting atherosclerosis. Gao et al. (2016) found that the exosomes from bone marrow dendritic cells increased endothelial inflammation by mediating tumor necrosis factor (TNF-α) release *via* the NF-κB pathway. Exosomes harboring miR-16 and miR-21 can inhibit the NF-κB pathway, thereby inhibiting the endothelial inflammatory reaction induced by TNF-α, which can retard the progress of atherosclerosis (Li et al., 2019). Therefore, exosomal miR-146a and miR-223 may be the molecular targets for treatment of atherosclerosis, and regulation of the NF-κB pathway may be potentially used for retarding atherosclerosis.

### 6.2 Role of exosomal miRNAs in myocardial injury and infarction

As atherosclerosis aggravates, the coronary artery narrows, and the coronary blood flow is suddenly interrupted. As a result, the downstream blood flow is blocked and the myocardial supply and demand balance is disrupted. The inflammatory substances from the atherosclerotic plaque destroy the integrity of fiber caps. Blocking causes myocardial damage, which in turn induces cardiomyocyte apoptosis. Myocardial infarction is one of the main causes of heart remodeling and failure and is associated with high incidence and death rates (Lazar et al., 2018). Coronary angioplasty can repair the damaged myocardium after myocardial infarction to only a certain extent. Recently, many reports have shown that exosomal miRNAs can regulate the damage and apoptosis of cardiomyocytes, participating in the process of myocardial infarction and finally promoting intercellular communication between cells (Cheng et al., 2020). Thus, they play important roles in the pathophysiology of myocardial infarction and affect the diagnosis and treatment of the disease.

During acute myocardial infarction, the cardiomyocyte-derived exosomes are in an oxygen-deficient state and contain miRNAs, such as miR-17 and miR-324. Li et al. (2019) found that miR-17 can activate the PI3K/Akt and TIMP1/2→MMP9 pathways. This reduces the lesion area of myocardial infarction and enhances the cardiac response to a certain extent. At the same time, they can affect metalloprotease expression, induce the formation of capillaries, and enhance repair and tolerance to hypoxia (Hu et al., 2019; Pan et al., 2019; Sun et al., 2019). Han et al. (2020) found that miR-324 induced apoptosis and inhibited cell proliferation by regulating the expression of caspase-3 and p-P38-MAPK. In addition, by regulating the TNF-α and NF-κB signaling pathways and the protein levels of TNF-α, miR-324 can alleviate the damage caused by cardiomyocyte hypoxia (Huang S et al., 2021). Furthermore, some exosomes can improve cardiac function after cardiomyocyte infarction, while some may also increase myocardial injury after myocardial infarction (Mao et al., 2019). In mouse models of myocardial infarction, Wang et al. (2017) observed that the exosomal miR-155 derived from macrophages was significantly upregulated. The exosomal form of this miRNA inhibits fibroblast proliferation and promotes cardiac inflammatory response during myocardial infarction. In experiments where exosomal miR-155 was inhibited or knocked out, the progression of myocardial infarction was studied more deeply.

Studies have shown that exosomal miRNAs are highly related to the progression of myocardial infarction. The number of molecules incorporated by the exosomes released by cardiomyocytes varies with the changes in cell culture conditions. Currently, troponin is the commonly used index of cardiomyocyte damage; however, for acute patients, the troponin level peaks 12 h after the attack (Youn et al., 2019). In contrast, some highly specific miRNAs, such as miR-1, miR-208a, and miR-192, appear rapidly in the blood after the attack (Li et al., 2018). In particular, the expression of miR-1 decreases after myocardial infarction, while the area of myocardial infarction increases. At the same time, the level of miR-1 in the patient’s serum decreases significantly (Wang S. et al., 2019). Moreover, the levels of miR-208a change significantly 4 h after acute myocardial infarction (Vanni et al., 2017). Therefore, considering the specific expression of the exosomes after myocardial infarction, as well as their ability to repair the damaged myocardium after myocardial infarction, the prospects of using exosomes in the diagnosis, treatment, and prognosis of myocardial infarction appear promising.

### 6.3 Role of exosomal miRNAs in heart failure

Heart failure is a complex which is the final outcome of cardiovascular diseases, and the incidence and death rate associated with which are higher in the elderly. Despite the current treatment regimen, the 5-year survival rate is still less than 50% (Snipelisky et al., 2019). Hence, the treatment of heart failure is a global public health issue. Ventricular remodeling, which includes cardiomyocyte hypertrophy, interstitial fibrosis, and activation of the renin–angiotensin system, is the basic pathological manifestation of heart failure. The compensation performance of cardiac hypertrophy leads to cardiac blood filling and discharge. Clinical symptoms may be absent or may manifest as asthma, edema, and other obvious dysfunctions in severe cases. Several studies have shown that exosomes play an important part in the diagnosis and treatment of heart failure, especially in those without any symptoms (Xue et al., 2020). At the same time, miRNAs in the exosomes can affect the pathological process to mediate ventricular remodeling. In particular, miRNAs can modulate cell proliferation and participate in cardiomyocyte stress. In addition, they can change the local microenvironment and promote vascular regeneration and reformation of damaged myocardial tissue. Hence, exosomes and miRNAs may affect the treatment of heart failure.

Exosomes secrete many types of miRNAs, among which, miR-21 has been studied in cases of heart failure. Activation of the renin–angiotensin system is one of the mechanisms of heart failure. Angiotensin IIS is significantly upregulated, which can lead to heart failure. Changes in the level of miR-21 can inhibit myocardial hypertrophy and simultaneously delay the myocardial hypertrophy caused by angiotensin II (Cheng et al., 2022). Qiao et al. (2019) compared the matrix cells of healthy cardiac tissue with those from the cardiac tissue of patients with heart failure and found that the level of miR-21 in the healthy cardiac tissue was higher than that in the diseased tissue. miR-21 regulates the procedural cell deaths caused by apoptosis in cardiomyocytes. Further studies have confirmed that miR-21 can promote angiogenesis and cardiomyocyte survival by inhibiting the activity of phosphatase tension protein (PTEN) and enhancing the activity of the protein kinase B (PKB) *in vivo* (Zhu et al., 2019). Therefore, it was believed that an increase in the expression of miR-21 in patients with heart failure may indicate conduciveness to the treatment of patients with heart failure. However, we found that excessive exosomes may also promote cardiac hypertrophy (Nie et al., 2018). Therefore, the therapeutic effect of exosomes and miRNAs has to be extensively assessed *via* detailed experimental verification.

Methods of diagnosing heart failure are also constantly improving. Currently, the most popular biomarkers of heart failure include BNP and N-terminal proBNP (NT-proBNP), which have higher sensitivity (Rørth et al., 2020). However, the specificity of these markers is limited, and age, default state of an individual, and other diseases such as right ventricular lesions and myocardial infarction may affect interpretation based on these markers, as a result of which diagnosis of heart failure may be influenced or even delayed to some extent. Studies have shown differences in expression levels of exosomes and miRNAs in the plasma of patients with heart failure. For example, the miR-146a level increased, while miR-21, miR-425, and miR-744 levels decreased. In addition, the exosomal miRNAs in vascular endothelial fibroblasts are inhibited; hence, the expression levels of miRNAs in the circulatory system can reflect the condition of cardiac fibroblasts (Ma et al., 2018). Emanuel et al. (2016) have shown that the expression of miR-146a in the exosomes of patients with heart failure correlated well with the level of cardiac troponin I (cTn-I). Wu et al. (2018) have observed a correlation between the miR92b-5p level and cardiac atrioventricular size in echocardiography. This suggested that the level of miR92b-5p increased with a decrease in left ventricle function. Therefore, use of the combination of miRNAs and other diagnostic methods, such as echocardiography or laboratory tests, may be one of the directions in translational research on exosomes.

### 6.4 Role of exosomal miRNAs in aortic dissection

Aortic dissection is one of the most dangerous cardiovascular diseases. Its rapid onset, high mortality rate, and poor prognosis severely affect the quality of life of the patients. Currently, in addition to symptom-based diagnosis, diagnosis of aortic dissection relies on the inspection of the aorta computed tomography angiography, although it is time-consuming, expensive, and associated with risk of kidney damage. At the same time, because of fluctuation in blood pressure during transportation, the risk of aortic dissection or rupture increases. Therefore, a highly sensitive, specific, and time- and effort-saving diagnostic method is required.

Phenotypic transformation of vascular smooth muscle cells in the middle aortic layer and its role in the pathogenesis of aortic dissection are being investigated. In addition, how the miRNAs incorporated by exosomes affect the phenotypic transformation of vascular smooth muscle cells is being actively researched. At present, the expression of at least five miRNAs, including miR-155, has been found to be significantly reduced in patient serum. Hsa-miR-155–5p regulates the expression of target genes and those related to the smooth muscle cells *via* the NF-κB signaling pathway. In this way, it induces phenotypic transformation and changes cell morphology, proliferation, and migration (Choi et al., 2018). Hsa-miR-26a-5p regulates BMP/SMAD1 signaling to targeted genes *via* receptor activation factors and tissue growth factors (Aschacher et al., 2022). Another study found that the miR-320 series was also involved in the migration and proliferation of cells, which in turn affected the function of endothelial cells and smooth muscle cells in the aorta (Liao et al., 2018). In the presence of high shear stress, hsa-miR-320d promotes apoptosis of cells by inhibiting the proliferation and migration of smooth muscle cells and endothelial cells, thereby maintaining the tension of the blood vessel wall (Ji et al., 2019). The research found that the level of miR-146a-5p in the plasma of patients with aortic dissection was significantly higher than that in healthy blood vessels (Xue et al., 2019). Furthermore, miR-134–5p is a key regulator that controls the phenotypic conversion and migration of smooth muscle cells and simultaneously participates in the progression of aortic dissection (Wang ZF. et al., 2019). In addition, miR-223–3p derived from the platelets acts as an endocrine genetic signal that reduces the blood vessel density after it enters the endothelium and vascular smooth muscle cells (Wang H. et al., 2019; Aschacher et al., 2021). Studies have shown that TGFB2 is the target of miR-599 in smooth muscle cells, while SEMA and TWIST2 are the target genes of hsa-miR-182–5p in smooth muscle cells. They prevent intima formation by inhibiting proliferation and migration during aortic dissection, (Mimler et al., 2019; Wu et al., 2019). In addition, miR-145, which is mainly expressed in vascular smooth muscle cells, is one of the core factors regulating vascular phenotype conversion. Therefore, if exosomal miRNAs that are specifically expressed in the aortic dissection are identified, there will be a breakthrough in the diagnosis, treatment, and prevention of aortic dissection.

### 6.5 Role of exosomal miRNAs in myocardial fibrosis

Myocardial fibrosis involves alterations in normal tissue structure due to changes in myocardial collagen fiber dynamics, including excessive accumulation and increase in the concentration of the fiber, or changes in the fiber components. Normal cardiac fiber cells secrete the extracellular matrix that provides a stable stent structure for the heart. Myocardial cells undergo necrosis during myocardial infarction. The myocardial fibroblasts are activated to cardiac fibroblasts, which then increase fiber synthesis. Fiber synthesis results in the formation of scar tissue, which replaces the original myocardial cells. Studies have shown that the inflammatory factors activated by the death of cardiomyocytes are closely related to the necrosis of activated myocardial fibroblasts. Some exosomal miRNAs may be associated with the activity of inflammatory factors, and they may affect the activation and hyperplasia of myocardial fibroblasts (Hohn et al., 2021). Furthermore, some of the exosomal miRNAs can perform biological functions such as resistance to cardiac apoptosis and reduction in collagen production, thereby reducing myocardial fibrosis to some extent (Ferguson et al., 2018).

The level of miR-21 can affect the hypertrophic growth of cardiomyocytes. Possibly, the exosomes that contain miR-21 can reduce apoptosis of myocardial cells and endothelial cells to a certain extent, thereby reducing the activation of myocardial fibroblasts into cardiac fibroblasts. While some cardiomyocytes undergo necrosis, the exosomes expressing low levels of miR-21–5p are released, following which they relay information *via* the phosphatase and tensin homolog/threonine kinase 1 (PTEN/AKT) and phosphatase pathways to induce near-normal cardiomyocyte apoptosis. At the same time, cardiomyocytes are constantly activating and proliferating into cardiac fibroblasts (Frangogiannis, 2019). In addition, apoptosis of cardiomyocytes was reduced when exosomes rich in miR-21–5p were co-cultured with cardiomyocytes, and the activation and hyperplasia of myocardial fibroblasts also declined accordingly. At the same time, the content of caspase-3 in cardiomyocytes also decreased. A study found that miR-19a-3p present in exosomes derived from endothelial cells can activate the AKT and extracellular-signal-regulated kinase (ERK) pathway, which can significantly reduce myocardial fibrosis (Gollmann-Tepeköylü et al., 2020). A similar function has been observed for other exosomal miRNAs, such as miR-294, miR-24, and miR-125b-5p (Moghaddam et al., 2019). A recent study showed that scar formation and inhibition of fibrosis after injecting exosomes rich in miR-146a or miR-21 into a mouse model differed considerably from those in the control group. Therefore, the inhibitory and therapeutic effects of exosomes and miRNAs on myocardial fibrosis require more in-depth investigations.

### 6.6 Role of exosomal miRNAs in ischemia–reperfusion

Ischemia–reperfusion injury refers to the injury after reperfusion treatment using balloon, stent, and coronary artery bypass grafting. As a result of these procedures, certain events such as reperfusion arrhythmia, myocardial stunning, and microvascular dysfunction occur in the ischemic myocardium. These may further aggravate to myocardial injury. A large number of studies have shown that the miRNAs present in exosomes can play a positive protective role by regulating various processes, such as apoptosis, inflammation, autophagy, and oxidative stress, to reduce ischemia–reperfusion injury (Bei et al., 2017; Zhang M. et al., 2019).

The most fundamental way of improving ischemia–reperfusion injury is to reduce cardiomyocyte death and cardiac dysfunction. Hou Z. et al. (2019) found that in the hypoxia reoxygenation model, the expression of caspase-9 and protein JNK2 decreased significantly because of the upregulation of miR-342–5p. Release of lactate dehydrogenase was inhibited, which increased cell viability. At the same time, Chen et al. (2017) have shown that the level of miR-30a in the exosomes increased after ischemia–reperfusion due to induction of hypoxia-inducible factor-1α (HIF-1α), whereas the activity of autophagy-associated proteins, Atg12 and beclin-1, and cardiomyocyte death decreased accordingly.

In addition to reducing cardiomyocyte death, the inflammatory response also plays an important role in ameliorating ischemia–reperfusion injury. In the presence of ischemia–reperfusion, monocytes in peripheral blood and blood circulation gradually gathered at the damaged myocardium and transformed into M1 and M2 macrophages under the effect of differentiation promoting factors to promote inflammation or anti-inflammation (Shiraishi et al., 2016; Li et al., 2019). During the repair of myocardial injury in ischemia–reperfusion, Jia et al. (2017) found that completely inhibiting the formation of M1 macrophages was not ideal, while reducing the number of the M1 type and increasing that of M2 macrophages effectively alleviated ischemia–reperfusion injury. Hence, regulating the level of M2 macrophages is critical for reducing ischemia–reperfusion injury. At the same time, Wen et al. (2018) showed that injecting exosomes derived from stem cells highly expressing miRNA-181a into the ischemia–reperfusion animal models promoted TREG polarization of peripheral blood mononuclear cells because of reduction in c-Fos protein level. Therefore, the targeting ability of exosomes and the immunosuppressive effect of the miRNAs harbored by the exosomes can be utilized for alleviating ischemic reperfusion injury. In addition, studies have shown that miR-148a can inhibit the activation of the NLRP3 inflammasome, mainly by lowering the expression of thioredoxin and its interacting protein and intervening in the TLR4/NF-κB signal pathway.

Furthermore, studies have shown that oxidative stress also contributes to ischemia–reperfusion injury and that oxidation stress is closely related to the exosomal miRNAs. Multiple reports have shown that miR-150, miR-21, miR-126, and other exosomal miRNAs, which participate in the ischemia–reperfusion injury procession, were induced by oxidation stress (Kura et al., 2020). In summary, exosomes are important carriers of information that can be exchanged between cells because of their good biocompatibility and high stability. The miRNAs secreted from the stem cells’ exosomes can be absorbed by the cardiac cells directly and can be used for the treatment of ischemia–reperfusion injury. Thus, exosomal miRNAs will be the next generation of therapeutics in the future (Zhou H et al., 2019; He et al., 2020).

### 6.7 Role of exosomal miRNAs in atrial fibrillation

The onset of atrial fibrillation is closely related to changes in electrophysiology and the function of ion channels, especially the calcium and kalium channels. Mutations in genes related to the potassium ion channel and occurrence of ectopic excitatory focus contribute to atrial fibrillation (Yao et al., 2021). Furthermore, atrial fibrillation is related to the size of the atrioventricular block and the degree of fibrosis. As mentioned previously, miRNAs present in exosomes regulate myocardial fibrosis; therefore, miRNAs are also necessarily related to atrial fibrillation.

Terentyev et al. (2009) have shown that miR-1 present in the exosomes is related to the opening of the atrial muscle voltage-gated calcium and kalium. The kalium opens when miR-1 expression decreases, which may promote the occurrence of atrial fibrillation. However, when the expression of miR-1 increases, more calcium ions flow into the atrial cells and promote atrial fibrillation. Park et al. (2017) observed that exosomes expressing miR-1 can reduce myocardial systolic dysfunction caused by atrial fibrillation in experimental models. Atrial fibrillation can shorten the duration of the action potential and lead to loss of the L-type calcium ion channel and calcium ion transient amplitude, whereas the modified exosomes effectively prevented this change. Lu et al. (2010) demonstrated that miR-328 was significantly upregulated in patients with atrial fibrillation, which caused electrical remodeling by the calcium ion channel encoded by the L-type genes, CACNAIC and CACNBI, to promote atrial fibrillation. Knocking down the expression of this miRNA reduced the occurrence of atrial fibrillation. Zhao et al. (2016) found that miR-29a-3p and the CACNA1C pathway were negatively regulated. As miR-29a-3p exerts strong and direct inhibitory effects on atrial muscles, modified exosomes expressing miR-29a-3p may be used as a therapeutic for atrial fibrillation.

Using microarray analysis, Mun et al. (2019) found that the serum levels of exosomal miRNAs, such as miR-320d, miR-486–5p, miR-107, and miR-103a-3p, were significantly increased in cases of atrial fibrillation. Results of the multivariable analysis revealed an independent correlation between certain miRNAs present in exosomes and atrial fibrillation. In addition, Wang J. et al. (2019) and Liu et al. (2020) found that the expression of more than 39 miRNAs in the exosomes of patients with atrial fibrillation differed from that in healthy controls, including 21 significantly upregulated miRNAs, two of them are miR-223–5p and miR-223–3p, which are related to the heart, and the expression was verified using qPCR. At the same time, two miRNAs were significantly downregulated, namely, miR-3126–5p and miR-27b-3p. A study showed that the expression of miR-320d in cardiomyocytes of patients with atrial fibrillation increases apoptosis and inhibits cell viability *via* the STAT3 pathway. Transmission of miR-320d mimics *via* stem cells can change their effects on cardiomyocytes. Another study showed that injection of exosomes that expressed miR-27b-3p into experimental models reduced the activity of the Wnt/β-catenin pathway to control atrial fibrillation (Lv et al., 2019).

In addition, studies have revealed that exosomal miRNAs are associated with the severity of atrial fibrillation. Comparison of patients with paroxysmal atrial fibrillation, persistent atrial fibrillation, and permanent atrial fibrillation indicated that the increase and decrease in the expression of miRNAs were inconsistent. In patients with persistent atrial fibrillation, the expression of some miRNAs such as miRNA-103a, miRNA-107, miRNA-320d, and miRNA-486 increased. However, their expression in patients with paroxysmal atrial fibrillation decreased. In summary, these studies provide insights regarding the prevention, mechanism of action, and severity of atrial fibrillation, as well as its prognosis and therapy (Huang Z et al., 2021). Gradually, as more miRNAs are discovered, the potential of therapeutic intervention with exosomes as carriers will amplify.

## 7 Role of exosomal miRNAs in other vascular diseases

In addition, the application of exosomal miRNA in other vascular diseases is also the international research highlights, which includes vascular Alzheimer’s disease (AD). At present, a large number of studies showed that one of the typical pathological changes of AD is the deposition of amyloid beta (Aβ). Aβ is produced during the amyloid precursor protein (APP) hydrolysis by β-secretory enzymes and γ-secretory enzymes (Yuyama and Igarashi, 2017). Research showed that the exosomal miRNAs are sensitive to the hydrolysis of Aβ. At the same time, it can penetrate blood barriers freely and directly act on the central nervous system. In AD patients, dysregulated miRNAs such as miR-101–3p and miR-106b could affect the expression of APP and other proteins. Then, they produced Aβ to aggravate the progress of AD (Iwata et al., 2014; Lin et al., 2018; Liu et al., 2014). In addition, dysregulated miR-132 and miR-212 could affect the synthesis and phosphorylation of tau protein, which affected the pathological process of AD. Smith et al. (2015) showed that miR-132 deficiency was relevant to autophagy dysfunction and long-term memory loss. After miR-132 is transferred into the neuron through exosomes, it could significantly improve memory impairment. In summary, many studies found that exosomal miRNA played an active role during the treatment of AD. In addition, exosomes and their miRNAs are stable, which could freely penetrate blood barriers and exist in the peripheral blood. They are simpler and more sensitive to the diagnosis of AD than MRI or cerebrospinal fluid markers, which have great potential in the early diagnosis and prevention of AD.

## 8 Expectations

The role of exosomal miRNAs in the development of cardiovascular diseases is beginning to be understood. In future, exosomes will have extensive application prospects in the diagnosis, treatment, and prevention of cardiovascular diseases. At the same time, research on exosomes is still in the preliminary stage, and the limitations of such studies are also obvious. The complex and expensive purification technology of exosomes hinders their universal application. For certain diseases, the role of exosomal miRNAs remains a double-edged sword, which can aggravate the progress of the diseases if not properly controlled. Targeted transport of exosomal miRNAs also has to be developed. With advancements in medical development and research, we believe that exosomal miRNAs will be used for improving public health and that they will play critical roles in the prevention, diagnosis, and treatment of diseases.

## Data Availability

The raw data supporting the conclusions of this article will be made available by the authors, without undue reservation.

## Abbreviations

NF-κB, nuclear factor kappa-B; TNF-α, tumor necrosis factor alpha; TM4SF, transmembrane 4 superfamily; FACS, fluorescent-activated cell sorting; PTEN, phosphatase tension protein; PKB, protein kinase B; NT-proBNP, N-terminal proBNP; cTn-I, cardiac troponin I.

## References

[B1] Aghabozorgi A. S. Ahangari N. Eftekhaari T. E. Torbati P. N. Bahiraee A. Ebrahimi R. (2019). Circulating exosomal miRNAs in cardiovascular disease pathogenesis: New emerging hopes. J. Cell. Physiol. 234 (12), 21796–21809. 10.1002/jcp.28942 31273798

[B2] Aschacher T. Aschacher O. Schmidt K. Enzmann F. K. Eichmair E. Winkler B. (2022). The role of telocytes and telocyte-derived exosomes in the development of thoracic aortic aneurysm. Int. J. Mol. Sci. 23 (9), 4730. 10.3390/ijms23094730 35563123 PMC9099883

[B3] Aschacher T. Schmidt K. Aschacher O. Eichmair E. Baranyi U. Winkler B. (2021). Telocytes in the human ascending aorta: Characterization and exosome-related KLF-4/VEGF-A expression. J. Cell. Mol. Med. 25 (20), 9697–9709. 10.1111/jcmm.16919 34562312 PMC8505852

[B4] Bei Y. Chen T. Banciu D. D. Cretoiu D. Xiao J. (2017). Circulating exosomes in cardiovascular diseases. Adv. Exp. Med. Biol. 998, 255–269. 10.1007/978-981-10-4397-0_17 28936745

[B5] Beltrami C. Besnier M. Shantikumar S. Shearn A. I. U. Rajakaruna C. Laftah A. (2017). Human pericardial fluid contains exosomes enriched with cardiovascular-expressed MicroRNAs and promotes therapeutic angiogenesis. Mol. Ther. 25 (3), 679–693. 10.1016/j.ymthe.2016.12.022 28159509 PMC5363195

[B6] Chen G. H. Xu J. Yang Y. J. (2017). Exosomes:promising sacks for treating ischemicheart disease. Am. J. Physiol. Heart Circ. Physiol. 313, H508–H523. 10.1152/ajpheart.00213.2017 28646026

[B7] Cheng G. Zhu D. Huang K. Caranasos T. G. (2022). Minimally invasive delivery of a hydrogel-based exosome patch to prevent heart failure. J. Mol. Cell. Cardiol. 169, 113–121. 10.1016/j.yjmcc.2022.04.020 35523270

[B8] Cheng H. Chang S. Xu R. Chen L. Song X. Wu J. (2020). Hypoxia-challenged MSC-derived exosomes deliver miR-210 to attenuate post-infarction cardiac apoptosis. Stem Cell. Res. Ther. 11 (1), 224. 10.1186/s13287-020-01737-0 32513270 PMC7278138

[B9] Choi S. Park M. Kim J. Park W. Kim S. Lee D. K. (2018). TNF-α elicits phenotypic and functional alterations of vascular smooth muscle cells by miR-155-5p-dependent down-regulation of cGMP-dependent kinase 1. J. Biol. Chem. 293 (38), 14812–14822. 10.1074/jbc.RA118.004220 30104414 PMC6153283

[B10] Chuppa S. Liang M. Liu P. Liu Y. Casati M. C. Cowley A. W. (2018). MicroRNA-21 regulates peroxisome proliferator-activated receptor alpha, a molecular mechanism of cardiac pathology in Cardiorenal Syndrome Type 4. Kidney Int. 93 (2), 375–389. 10.1016/j.kint.2017.05.014 28760335 PMC5787401

[B11] Ding L. Yang X. Gao Z. Effah C. Y. Zhang X. Wu Y. (2021). A holistic review of the state-of-the-art microfluidics for exosome separation: An overview of the current status, existing obstacles, and future outlook. Small 17 (29), e2007174. 10.1002/smll.202007174 34047052

[B12] Doyle L. M. Wang M. Z. (2019). Overview of extracellular vesicles, their origin, composition, purpose, and methods for exosome isolation and analysis. Cells 8 (7), 727. 10.3390/cells8070727 PMC6678302 31311206

[B13] Edgar J. R. (2016). Q&A: What are exosomes, exactly? BMC Biol. 14, 46. 10.1186/s12915-016-0268-z 27296830 PMC4906597

[B14] Emanueli C. Shearn A. I. Laftah A. Fiorentino F. Reeves B. C. Beltrami C. (2016). Coronary artery-bypass-graft surgery increases the plasma concentration of exosomes carrying a cargo of cardiac MicroRNAs: An example of exosome trafficking out of the human heart with potential for cardiac biomarker discovery. PLoS One 11 (4), e0154274. 10.1371/journal.pone.0154274 27128471 PMC4851293

[B15] Ferguson S. W. Wang J. Lee C. J. Liu M. Neelamegham S. Canty J. M. (2018). The microRNA regulatory landscape of MSC-derived exosomes: A systems view. Sci. Rep. 8 (1), 1419. 10.1038/s41598-018-19581-x 29362496 PMC5780426

[B16] Frangogiannis N. G. (2019). Cardiac fibrosis: Cell biological mechanisms, molecular pathways and therapeutic opportunities. Mol. Asp. Med. 65, 70–99. 10.1016/j.mam.2018.07.001 30056242

[B17] Gao W. Liu H. Yuan J. Wu C. Huang D. Ma Y. (2016). Exosomes derived from mature dendritic cells increase endothelial inflammation and atherosclerosis via membrane TNF-α mediated NF-κB pathway. J. Cell. Mol. Med. 20 (12), 2318–2327. 10.1111/jcmm.12923 27515767 PMC5134386

[B18] Gollmann-Tepeköylü C. Pölzl L. Graber M. Hirsch J. Nagele F. Lobenwein D. (2020). miR-19a-3p containing exosomes improve function of ischaemic myocardium upon shock wave therapy. Cardiovasc. Res. 116 (6), 1226–1236. 10.1093/cvr/cvz209 31410448

[B19] Han X. Chen X. Han J. Zhong Y. Li Q. An Y. (2020). MiR-324/SOCS3 Axis protects against hypoxia/reoxygenation-induced cardiomyocyte injury and regulates myocardial ischemia via TNF/NF-κB signaling pathway. Int. Heart J. 61 (6), 1258–1269. 10.1536/ihj.19-687 33191336

[B20] He N. Zhang Y. Zhang S. Wang D. Ye H. (2020). Exosomes: Cell-free therapy for cardiovascular diseases. J. Cardiovasc. Transl. Res. 13, 713–721. 10.1007/s12265-020-09966-7 32333198

[B21] Helwa I. Cai J. Drewry M. D. Zimmerman A. Dinkins M. B. Khaled M. L. (2017). A comparative study of serum exosome isolation using differential ultracentrifugation and three commercial reagents. PLoS One 12 (1), e0170628. 10.1371/journal.pone.0170628 28114422 PMC5256994

[B22] Heo J. Kang H. (2022). Exosome-based treatment for atherosclerosis. Int. J. Mol. Sci. 23 (2), 1002. 10.3390/ijms23021002 35055187 PMC8778342

[B23] Hohn J. Tan W. Carver A. Barrett H. (2021). Roles of exosomes in cardiac fibroblast activation and fibrosis. Cells 10 (11), 2933. 10.3390/cells10112933 34831158 PMC8616203

[B25] Hou Z. Qin X. Hu Y. Zhang X. Li G. Wu J. (2019). Longterm exercise-derived exosomal miR-342-5p:a novel exerkine for cardioprotection. Circ. Res. 124 (9), 1386–1400. 10.1161/CIRCRESAHA.118.314635 30879399

[B26] Hu H. Wang B. Jiang C. Li R. Zhao J. (2019). Endothelial progenitor cell-derived exosomes facilitate vascular endothelial cell repair through shuttling miR-21-5p to modulate Thrombospondin-1 expression. Clin. Sci. 133 (14), 1629–1644. 10.1042/CS20190188 31315970

[B27] Huang L. H. Rau C. Wu S. Wu Y. Tsai C. Ln C. (2021). Identification and characterization of hADSC-derived exosome proteins from different isolation methods. J. Cell. Mol. Med. 25 (15), 7436–7450. 10.1111/jcmm.16775 34235869 PMC8335681

[B28] Huang S. Deng Y. Xu J. Liu J. Liu L. Fan C. (2021). The role of exosomes and their cargos in the mechanism, diagnosis, and treatment of atrial fibrillation. Front. Cardiovasc. Med. 8, 712828. 10.3389/fcvm.2021.712828 34395566 PMC8355361

[B29] Huang Z. Zhao D. Wang Y. Li X. Li J. Han J. (2021). C1q/TNF-related protein 9 decreases cardiomyocyte hypoxia/reoxygenation-induced inflammation by inhibiting the TLR4/MyD88/NF-κB signaling pathway. Exp. Ther. Med. 22 (4), 1139. 10.3892/etm.2021.10573 34504585 PMC8393267

[B30] Iwata A. Nagata K. Hatsuta H. Takuma H. Bundo M. Iwamoto K. (2014). Altered CpG methylation in sporadic Alzheimer's disease is associated with APP and MAPT dysregulation. Hum. Mol. Genet. 23 (3), 648–656. 10.1093/hmg/ddt451 24101602

[B120] Jeppesen D. K. Hvam M. L. Primdahl-Bengtson B. Boysen A. T. Whitehead B. Dyrskjøt L. (2014). Comparative analysis of discrete exosome fractions obtained by differential centrifugation. Extracell Vesicles. 3, 25011. 10.3402/jev.v3.25011 PMC4224706 25396408

[B31] Ji Q. Wang Y. L. Xia L. M. Yang Y. Wang C. S. Mei Y. Q. (2019). High shear stress suppresses proliferation and migration but promotes apoptosis of endothelial cells co-cultured with vascular smooth muscle cells via down-regulating MAPK pathway. J. Cardiothorac. Surg. 14 (1), 216. 10.1186/s13019-019-1025-5 31831023 PMC6909635

[B32] Jia C. Chen H. Wei M. Chen X. Zhang Y. Cao L. (2017). Gold nanoparticle- based miR155 antagonist macrophage delivery restores the cardiac function in ovariectomized diabetic mouse model. Int. J. Nanomedicine 12, 4963–4979. 10.2147/IJN.S138400 28744126 PMC5513843

[B33] Johnstone R. M. Adam M. Hammond J. R. Orr L. Turbide C. (1987). Vesicle formation during reticulocyte maturation Association of plasma membrane activities with released vesicles ( exosomes). J. Biol. Chem. 262, 9412–9420. 10.1016/s0021-9258(18)48095-7 3597417

[B34] Jung M. K. Mun J. Y. (2018). Sample preparation and imaging of exosomes by transmission electron microscopy. J. Vis. Exp. 131, 56482. 10.3791/56482 PMC5908436 29364263

[B35] Kogure A. Yoshioka Y. Ochiya T. (2020). Extracellular vesicles in cancer metastasis: Potential as therapeutic targets and materials. Int. J. Mol. Sci. 21 (12), 4463. 10.3390/ijms21124463 PMC7352700 32585976

[B36] Kumar D. Narang R. Sreenivas V. Rastogi V. Bhatia J. Saluja D. (2020). Circulatory miR-133b and miR-21 as novel biomarkers in early prediction and diagnosis of coronary artery disease. Genes. 11 (2), E164. 10.3390/genes11020164 32033332 PMC7073535

[B37] Kura B. Szeiffova Bacova B. Kalocayova B. Sykora M. Slezak J. (2020). Oxidative stress-responsive MicroRNAs in heart injury. Int. J. Mol. Sci. 21 (1), 358. 10.3390/ijms21010358 PMC6981696 31948131

[B38] Lazar E. Benedek T. Korodi S. Rat N. Lo J. Benedek I. (2018). Stem cell-derived exosomes - an emerging tool for myocardial regeneration. World J. Stem Cells 10 (8), 106–115. 10.4252/wjsc.v10.i8.106 30190780 PMC6121000

[B39] Lazar S. Wurtzel J. G. T. Chen X. Ma P. Goldfinger L. E. (2021). High-efficiency unassisted transfection of platelets with naked double-stranded miRNAs modulates signal-activated translation and platelet function. Platelets 32 (6), 794–806. 10.1080/09537104.2020.1809642 32838617 PMC8415128

[B40] Li H. Liao Y. Gao L. Zhuang T. Huang Z. Zhu H. (2018). Coronary serum exosomes derived from patients with myocardial ischemia regulate angiogenesis through the miR-939-mediated nitric oxide signaling pathway. Theranostics 8 (8), 2079–2093. 10.7150/thno.21895 29721064 PMC5928872

[B41] Li J. W. Wei L. Han Z. Chen Z. (2019). Mesenchymal stromal cells-derived exosomes alleviate ischemia/reperfusion injury in mouse lung by transporting anti-apoptotic miR-21-5p. Eur. J. Pharmacol. 852, 68–76. 10.1016/j.ejphar.2019.01.022 30682335

[B42] Li P. Kaslan M. Lee S. H. Yao J. Gao Z. (2017). Progress in exosome isolation techniques. Theranostics 7 (3), 789–804. 10.7150/thno.18133 28255367 PMC5327650

[B43] Li W. Wang Q. (2021). Recent progress in the research of exosomes and Dscam regulated crab antiviral immunity. Dev. Comp. Immunol. 116, 103925. 10.1016/j.dci.2020.103925 33217412

[B44] Li X. Li X. Lin J. Sun X. Ding Q. (2019). Exosomes derived from low intensity pulsed ultrasound-treated dendritic cells suppress tumor necrosis factor-induced endothelial inflammation. J. Ultrasound Med. 38 (8), 2081–2091. 10.1002/jum.14898 30561085

[B45] Liao M. Zou S. Bao Y. Jin J. Yang J. Liu Y. (2018). Matrix metalloproteinases are regulated by MicroRNA 320 in macrophages and are associated with aortic dissection. Exp. Cell. Res. 370 (1), 98–102. 10.1016/j.yexcr.2018.06.011 29908163

[B46] Lin S. Yu Z. Chen D. Wang Z. Miao J. Li Q. (2020). Progress in microfluidics-based exosome separation and detection technologies for diagnostic applications. Small 16 (9), e1903916. 10.1002/smll.201903916 31663295

[B47] Lin Y. Yao Y. Liang X. Shi Y. Kong L. Xiao H. (2018). Osthole suppresses amyloid precursor protein expression by up-regulating miRNA-101a-3p in Alzheimer's disease cell model. Zhejiang Da Xue Xue Bao Yi Xue Ban. 47 (5), 473–479. 10.3785/j.issn.1008-9292.2018.10.05 30693688 PMC10393713

[B48] Lindenbergh M. F. S. Koerhuis D. G. J. Borg E. G. F. van 't Veld E. M. Driedonks T. A. P. Wubbolts R. (2018). Bystander T-cells support clonal T-cell activation by controlling the release of dendritic cell-derived immune-stimulatory extracellular vesicles. Front. Immunol. 10, 448. 10.3389/fimmu.2019.00448 PMC6423080 30915085

[B49] Lindenbergh M. F. S. Stoorvogel W. (2018). Antigen presentation by extracellular vesicles from professional antigen-presenting cells. Annu. Rev. Immunol. 36, 435–459. 10.1146/annurev-immunol-041015-055700 29400984

[B50] Lindenbergh M. F. S. Wubbolts R. Borg E. G. F. van 't Veld E. M. Boes M. Stoorvogel W. (2020). Dendritic cells release exosomes together with phagocytosed pathogen; potential implications for the role of exosomes in antigen presentation. J. Extracell. Vesicles 9 (1), 1798606. 10.1080/20013078.2020.1798606 32944186 PMC7480536

[B51] Liu C. G. Song J. Zhang Y. Q. Wang P. C. (2014). MicroRNA-193b is a regulator of amyloid precursor protein in the blood and cerebrospinal fluid derived exosomal microRNA-193b is a biomarker of Alzheimer's disease. Mol. Med. Rep. 10 (5), 2395–2400. 10.3892/mmr.2014.2484 25119742

[B52] Liu L. Chen Y. Shu J. Tang C. E. Jiang Y. Luo F. (2020). Identification of microRNAs enriched in exosomes in human pericardial fluid of patients with atrial fibrillation based on bioinformatic analysis. J. Thorac. Dis. 12 (10), 5617–5627. 10.21037/jtd-20-2066 33209394 PMC7656334

[B53] Lu J. Wu J. Tian J. Wang S. (2018). Role of T cell-derived exosomes in immunoregulation. Immunol. Res. 66 (3), 313–322. 10.1007/s12026-018-9000-0 29804198

[B54] Lu M. Yuan S. Li S. Li L. Liu M. Wan S. (2019). The Exosome Derived biomarker in atherosclerosis and its clinical application. J. Cardiovasc. Transl. Res. 12, 68–74. 10.1007/s12265-018-9796-y 29802541

[B55] Lu Y. Zhang Y. Wang N. Pan Z. Gao X. Zhang F. (2010). MicroRNA- 328 contributes to adverse electrical remodeling in atrial fibrillation. Circulation 122 (23), 2378–2387. 10.1161/CIRCULATIONAHA.110.958967 21098446

[B56] Lv X. Li J. Hu Y. Wang S. Yang C. Li C. (2019). Overexpression of miR-27b-3p targeting Wnt3a regulates the signaling pathway of Wnt/β-Catenin and attenuates atrial fibrosis in rats with atrial fibrillation. Oxid. Med. Cell. Longev. 2019, 5703764. 10.1155/2019/5703764 31178968 PMC6501122

[B57] Ma T. Chen Y. Chen Y. Meng Q. Sun J. Shao L. (2018). MicroRNA-132, delivered by mesenchymal stem cell-derived exosomes, promote angiogenesis in myocardial infarction. Stem Cells Int. 2018, 3290372. 10.1155/2018/3290372 30271437 PMC6151206

[B58] Mao Q. Liang X. L. Zhang C. L. Pang Y. H. Lu Y. X. (2019). LncRNA KLF3-AS1 in human mesenchymal stem cell-derived exosomes ameliorates pyroptosis of cardiomyocytes and myocardial infarction through miR-138-5p/Sirt1 axis. Stem Cell. Res. Ther. 10 (1), 393. 10.1186/s13287-019-1522-4 31847890 PMC6918658

[B59] Mashouri L. Yousefi H. Aref A. R. Ahadi A. M. Molaei F. Alahari S. K. (2019). Exosomes: Composition, biogenesis, and mechanisms in cancer metastasis and drug resistance. Mol. Cancer 18 (1), 75. 10.1186/s12943-019-0991-5 30940145 PMC6444571

[B60] Mimler T. Nebert C. Eichmair E. Winter B. Aschacher T. Stelzmueller M. E. (2019). Extracellular matrix in ascending aortic aneurysms and dissections - what we learn from decellularization and scanning electron microscopy. PLoS One 14 (3), e0213794. 10.1371/journal.pone.0213794 30883576 PMC6422325

[B61] Moghaddam A. S. Afshari J. T. Esmaeili S. A. Saburi E. Joneidi Z. Momtazi-Borojeni A. A. (2019). Cardioprotective microRNAs: Lessons from stem cell- derived exosomal microRNAs to treat cardiovascular disease. Atherosclerosis 285, 1–9. 10.1016/j.atherosclerosis.2019.03.016 30939341

[B62] Momen-Heravi F. (2017). Isolation of extracellular vesicles by ultracentrifugation. Methods Mol. Biol. 1660, 25–32. 10.1007/978-1-4939-7253-1_3 28828645

[B63] Moon S. Shin D. W. Kim S. Lee Y. S. Mankhong S. Yang S. W. (2019). Enrichment of exosome-like extracellular vesicles from plasma suitable for clinical vesicular miRNA biomarker research. J. Clin. Med. 8 (11), 1995. 10.3390/jcm8111995 PMC6912341 31731761

[B64] Mun D. Kim H. Kang J. Y. Park H. Park H. Lee S. H. (2019). Expression of miRNAs in circulating exosomes derived from patients with persistent at rial fibrillation. FASEB J. 33 (5), 5979–5989. 10.1096/fj.201801758R 30753098

[B65] Nam G. H. Choi Y. Kim G. B. Kim S. Kim S. A. Kim I. S. (2020). Emerging prospects of exosomes for cancer treatment: From conventional therapy to immunotherapy. Adv. Mat. 32 (51), e2002440. 10.1002/adma.202002440 33015883

[B66] Nguyen M. A. Karunakaran D. Geoffrion M. Cheng H. S. Tandoc K. Perisic Matic L. (2018). Extracellular vesicles secreted by atherogenic macrophages transfer MicroRNA to inhibit cell migration. Arterioscler. Thromb. Vasc. Biol. 38 (1), 49–63. 10.1161/ATVBAHA.117.309795 28882869 PMC5884694

[B67] Nie X. Fan J. Li H. Yin Z. Zhao Y. Dai B. (2018). miR-217 promotes cardiac hypertrophy and dysfunction by targeting PTEN. Mol. Ther. Nucleic Acids 12, 254–266. 10.1016/j.omtn.2018.05.013 30195764 PMC6005806

[B68] O'Brien J. Hayder H. Zayed Y. Peng C. (2018). Overview of MicroRNA biogenesis, mechanisms of actions, and circulation. Front. Endocrinol. 9, 402. 10.3389/fendo.2018.00402 PMC6085463 30123182

[B69] Pan Q. Wang Y. Lan Q. Wu W. Li Z. Ma X. (2019). Exosomes derived from mesenchymal stem cells ameliorate hypoxia/reoxygenation-injured ECs via transferring MicroRNA-126. Stem Cells Int. 2019, 2831756. 10.1155/2019/2831756 31281371 PMC6589209

[B70] Park H. Park H. L. Kim H. E. Mun D. Cui S. Joung B. (2017). P1562Exosome derived from atrial fibrillation patients prevents proarrhythmic remodeling by suppressing autophagy in pacing induced tachycardia model. Eur. Heart J. 38, 1562. 10.1093/eurheartj/ehx502.p1562

[B71] Pathan M. Fonseka P. Chitti S. V. Kang T. Sanwlani R. Van Deun J. (2019). Vesiclepedia 2019: a compendium of RNA, proteins, lipids and metabolites in extracellular vesicles. Nucleic Acids Res. 47, D516–D519. 10.1093/nar/gky1029 30395310 PMC6323905

[B72] Powers J. C. Sabri A. Al-Bataineh D. Chotalia D. Guo X. Tsipenyuk F. (2020). Differential microRNA-21 and microRNA-221 upregulation in the biventricular failing heart reveals distinct stress responses of right versus left ventricular fibroblasts. Circ. Heart Fail. 13 (1), e006426. 10.1161/CIRCHEARTFAILURE.119.006426 31916447 PMC7006717

[B73] Qiao L. Hu L. Liu S. Zhang H. Ma H. Huang K. (2019). microRNA- 21- 5p dysregulation in exosomes derived from heart failure patients impairs regenerative potential. J. Clin. Invest. 129, 2237–2250. 10.1172/JCI123135 31033484 PMC6546482

[B74] Ragusa M. Barbagallo C. Statello L. Caltabiano R. Russo A. Puzzo L. (2015). miRNA profiling in vitreous humor, vitreal exosomes and serum from uveal melanoma patients: Pathological and diagnostic implications. Cancer Biol. Ther. 16 (9), 1387–1396. 10.1080/15384047.2015.1046021 25951497 PMC4622662

[B75] Ramirez M. I. Amorim M. G. Gadelha C. Milic I. Welsh J. A. Freitas V. M. (2018). Technical challenges of working with extracellular vesicles. Nanoscale 10 (3), 881–906. 10.1039/c7nr08360b 29265147

[B76] Rong L. Li R. Li S. Luo R. (2016). Immunosuppression of breast cancer cells mediated by transforming growth factor-β in exosomes from cancer cells. Oncol. Lett. 11 (1), 500–504. 10.3892/ol.2015.3841 26870240 PMC4727188

[B77] Rørth R. Jhund P. S. Yilmaz M. B. Kristensen S. L. Welsh P. Desai A. S. (2020). Comparison of BNP and NT-proBNP in patients with heart failure and reduced ejection fraction. Circ. Heart Fail. 13 (2), e006541. 10.1161/CIRCHEARTFAILURE.119.006541 32065760

[B78] Ruivo C. F. Adem B. Silva M. Melo S. A. (2017). The biology of cancer exosomes: Insights and new perspectives. Cancer Res. 77 (23), 6480–6488. 10.1158/0008-5472.CAN-17-0994 29162616

[B79] Shi C. Ulke-Lemée A. Deng J. Batulan Z. O'Brien E. R. (2019). Characterization of heat shock protein 27 in extracellular vesicles: A potential anti-inflammatory therapy. FASEB J. 33 (2), 1617–1630. 10.1096/fj.201800987R 30188755

[B80] Shi X. Xie X. Sun Y. He H. Huang H. Liu Y. (2020). Paeonol inhibits NLRP3 mediated inflammation in rat endothelial cells by elevating hyperlipidemic rats plasma exosomal miRNA-223. Eur. J. Pharmacol. 885, 173473. 10.1016/j.ejphar.2020.173473 32800809

[B81] Shiraishi M. Shintani Y. Shintani Y. Ishida H. Saba R. Yamaguchi A. (2016). Alternatively activated macrophages determine repair of the infarcted adult murine heart. J. Clin. Invest. 126 (6), 2151–2166. 10.1172/JCI85782 27140396 PMC4887176

[B82] Smith P. Y. Hernandez-Rapp J. Jolivette F. Lecours C. Bisht K. Goupil C. (2015). miR-132/212 deficiency impairs tau metabolism and promotes pathological aggregation *in vivo* . Hum. Mol. Genet. 24 (23), 6721–6735. 10.1093/hmg/ddv377 26362250 PMC4634376

[B83] Snipelisky D. Chaudhry S. P. Stewart G. C. (2019). The many faces of heart failure. Card. Electrophysiol. Clin. 11, 11–20. 10.1016/j.ccep.2018.11.001 30717842

[B84] Sun Y. Liu X. L. Zhang D. Liu F. Cheng Y. J. Ma Y. (2019). Platelet-derived exosomes affect the proliferation and migration of human umbilical vein endothelial cells via miR-126. Curr. Vasc. Pharmacol. 17 (4), 379–387. 10.2174/1570161116666180313142139 29532758

[B85] Szabo G. Momen-Heravi F. (2017). Extracellular vesicles in liver disease and potential as biomarkers and therapeutic targets. Nat. Rev. Gastroenterol. Hepatol. 14 (8), 455–466. 10.1038/nrgastro.2017.71 28634412 PMC6380505

[B86] Tang X. H. Guo T. Gao X. Y. Wu X. L. Xing X. F. Ji J. F. (2021). Exosome-derived noncoding RNAs in gastric cancer: Functions and clinical applications. Mol. Cancer 20 (1), 99. 10.1186/s12943-021-01396-6 34330299 PMC8323226

[B87] Taylor D. D. Gercel-Taylor C. (2008). MicroRNA signatures of tumor-derived exosomes as diagnostic biomarkers of ovarian cancer. Gynecol. Oncol. 110 (1), 13–21. 10.1016/j.ygyno.2008.04.033 18589210

[B88] Terentyev D. Belevych A. E. Terentyeva R. Martin M. M. Malana G. E. Kuhn D. E. (2009). miR-1 overexpression enhances Ca(2+) release and promotes cardiac arrhythmogenesis by targeting PP2A regulatory subunit B56alpha and causing CaMKII-dependent hyperphosphorylation of RyR2. Circ. Res. 104 (4), 514–521. 10.1161/CIRCRESAHA.108.181651 19131648 PMC4394868

[B89] Tschuschke M. Kocherova I. Bryja A. Mozdziak P. Angelova Volponi A. Janowicz K. (2020). Inclusion biogenesis, methods of isolation and clinical application of human cellular exosomes. J. Clin. Med. 9 (2), E436. 10.3390/jcm9020436 32041096 PMC7074492

[B90] Vanni I. Alama A. Grossi F. Dal Bello M. G. Coco S. (2017). Exosomes: A new horizon in lung cancer. Drug Discov. Today 22 (6), 927–936. 10.1016/j.drudis.2017.03.004 28288782

[B91] Vizoso F. J. Eiro N. Cid S. Schneider J. Perez-Fernandez R. (2017). Mesenchymal stem cell secretome: Toward cell-free therapeutic strategies in regenerative medicine. Int. J. Mol. Sci. 18, 1852. 10.3390/ijms18091852 PMC5618501 28841158

[B92] Wang C. Zhang C. Liu L. A X. Chen B. Li Y. (2017). Macrophage Derived miR 155 containing exosomes suppress fibroblast proliferation and promote fibroblast inflammation during cardiac injury. Mol. Ther. 25, 192–204. 10.1016/j.ymthe.2016.09.001 28129114 PMC5363311

[B93] Wang H. Lu Z. Zhao X. (2019f). Tumorigenesis diagnosis and therapeutic potential of exosomes in liver cancer. J. Hematol. Oncol. 12, 133. 10.1186/s13045-019-0806-6 31815633 PMC6902437

[B94] Wang J. Huang X. Xie J. Han Y. Huang Y. Zhang H. (2021). Exosomal analysis: Advances in biosensor technology. Clin. Chim. Acta. 518, 142–150. 10.1016/j.cca.2021.03.026 33811925

[B95] Wang J. Zhao C. Xiao J. (2019g). Exosomes in cardiovas-cular diseases and treatment: Experimental and clinical aspects. J. Cardiovasc. Transl. Res. 12 (1), 1–2. 10.1007/s12265-018-9860-7 30617761

[B96] Wang S. Min J. Yu Y. Yin L. Wang Q. Shen H. (2019c). Differentially expressed miRNAs in circulating exosomes between atrial fibrillation and sinus rhythm. J. Thorac. Dis. 11 (10), 4337–4348. 10.21037/jtd.2019.09.50 31737319 PMC6837955

[B97] Wang X. Chen Y. Zhao Z. Meng Q. Yu Y. Sun J. (2018). Engineered exosomes with ischemic myocardium targeting peptide for targeted therapy in myocardial infarction. J. Am. Heart Assoc. 7, e008737. 10.1161/JAHA.118.008737 30371236 PMC6201471

[B98] Wang X. Ding Y. Y. Chen Y. Xu Q. Q. Qian G. H. Qian W. G. (2019b). MiR-223-3p alleviates vascular endothelial injury by targeting IL6ST in kawasaki disease. Front. Pediatr. 7 (3), 288. 10.3389/fped.2019.00288 31396494 PMC6667785

[B99] Wang Y. Dong C. Q. Peng G. Y. Huang H. Y. Yu Y. S. Ji Z. C. (2019a). MicroRNA-134-5p regulates media degeneration through inhibiting VSMC phenotypic switch and migration in thoracic aortic dissection. Mol. Ther. Nucleic Acids 16 (2), 284–294. 10.1016/j.omtn.2019.02.021 30951965 PMC6446055

[B100] Wang Y. Ma W. Q. Zhu Y. Han X. Q. Liu N. (2018). Exosomes derived from mesenchymal stromal cells pretreated with advanced glycation end product-bovine serum albumin inhibit calcification of vascular smooth muscle cells. Front. Endocrinol. Lausanne) 9, 524. 30298051 10.3389/fendo.2018.00524 PMC6160580

[B101] Wang Z. F. Liao F. Wu H. Dai J. (2019d). Glioma stem cells-derived exosomal miR-26a promotes angiogenesis of microvessel endothelial cells in glioma. J. Exp. Clin. Cancer Res. 38 (1), 201. 10.1186/s13046-019-1181-4 31101062 PMC6525364

[B102] Wei H. Chen Q. Lin L. Sha C. Li T. Liu Y. (2021). Regulation of exosome production and cargo sorting. Int. J. Biol. Sci. 17 (1), 163–177. 10.7150/ijbs.53671 33390841 PMC7757038

[B103] Wen M. H. Gong Z. J. Huang C. H. Qiang L. MinXuan X. Luqiao W. (2018). Plasma exosomes induced by remote ischaemic preconditioning attenuate myocardial ischaemia/reperfusion injury by transferring miR-24. Cell. Death Dis. 9 (3), 320. 10.1038/s41419-018-0274-x 29476052 PMC5833738

[B104] Wu J. H. Zhou Y. F. Hong C. D. Chen A. Q. Luo Y. Mao L. (2019). Semaphorin-3A protects against neointimal hyperplasia after vascular injury. EBioMedicine 39 (1), 95–108. 10.1016/j.ebiom.2018.12.023 30579864 PMC6355729

[B105] Wu Y. Chen Y. C. Du Y. T. Tao J. Li W. Zhou Z. (2018). Circulating exosomal miR-92b-5p is a promising diagnostic biomarker of heart failure with reduced ejection fraction patients hospitalized for acute heart failure. J. Thorac. Dis. 10 (11), 6211–6220. 10.21037/jtd.2018.10.52 30622793 PMC6297406

[B106] Xue L. Luo S. Ding H. Liu Y. Huang W. Fan X. (2019). Upregulation of miR-146a-5p is associated with increased proliferation and migration of vascular smooth muscle cells in aortic dissection. J. Clin. Lab. Anal. 33 (4), e22843. 10.1002/jcla.22843 30779466 PMC6528573

[B107] Xue R. Tan W. Wu Y. Dong B. Xie Z. Huang P. (2020). Role of exosomal miRNAs in heart failure. Front. Cardiovasc. Med. 7, 592412. 10.3389/fcvm.2020.592412 33392270 PMC7773699

[B108] Yang D. Zhang W. Zhang H. Zhang F. Chen L. Ma L. (2020). Progress, opportunity, and perspective on exosome isolation - efforts for efficient exosome-based theranostics. Theranostics 10 (8), 3684–3707. 10.7150/thno.41580 32206116 PMC7069071

[B109] Yang W. Zou B. Hou Y. Yan W. Chen T. Qu S. (2019). Extracellular vesicles in vascular calcification. Clin. Chim. Acta. 499, 118–122. 10.1016/j.cca.2019.09.002 31493375

[B110] Yao Y. He S. Wang Y. Cao Z. Liu D. Fu Y. (2021). Blockade of exosome release suppresses atrial fibrillation by alleviating atrial fibrosis in canines with prolonged atrial pacing. Front. Cardiovasc. Med. 8, 699175. 10.3389/fcvm.2021.699175 34722652 PMC8553970

[B111] Youn S. W. Li Y. Kim Y. M. Sudhahar V. Abdelsaid K. Kim H. W. (2019). Modification of cardiac progenitor cell-derived exosomes by miR-322 provides protection against myocardial infarction through nox2-dependent angiogenesis. Antioxidants (Basel) 8 (1), 18. 10.3390/antiox8010018 PMC6356993 30634641

[B112] Yuyama K. Igarashi Y. (2017). Exosomes as carriers of Alzheimer's amyloid-ß. Front. Neurosci. 11, 229. 10.3389/fnins.2017.00229 28487629 PMC5403946

[B113] Zhang H. Lyden D. (2019). Asymmetric-flow field-flow fractionation technology for exomere and small extracellular vesicle separation and characterization. Nat. Protoc. 14 (4), 1027–1053. 10.1038/s41596-019-0126-x 30833697 PMC6733524

[B114] Zhang M. Zhu J. Qin X. Zhou M. Zhang X. Gao Y. (2019b). Cardioprotection of tetrahedral DNA nanostructures in myocardial ischemia-reperfusion injury. ACS Appl. Mat. Interfaces 11 (34), 30631–30639. 10.1021/acsami.9b10645 31382735

[B115] Zhang T. R. Huang W. Q. (2021). Angiogenic exosome-derived microRNAs: Emerging roles in cardiovascular disease. J. Cardiovasc. Transl. Res. 14 (5), 824–840. 10.1007/s12265-020-10082-9 33104961

[B116] Zhang Y. G. Song Y. Guo X. L. Miao R. Y. Fu Y. Q. Miao C. F. (2019a). Exosomes derived from oxLDL-stimulated macrophages induce neutrophil extracellular traps to drive atherosclerosis. Cell. Cycle 18 (20), 2674–2684. 10.1080/15384101.2019.1654797 31416388 PMC6773244

[B117] Zhao Y. Yuan Y. Qiu C. (2016). Underexpression of CACNA1C caused by overexpression of microRNA-29a underlies the pathogenesis of atrial fibrillation. Med. Sci. Monit. 22, 2175–2181. 10.12659/msm.896191 27341015 PMC4924888

[B118] Zheng D. Huo M. Li B. Wang W. Piao H. Wang Y. (2021). The role of exosomes and exosomal MicroRNA in cardiovascular disease. Front. Cell. Dev. Biol. 8, 616161. 10.3389/fcell.2020.616161 33511124 PMC7835482

[B24] Zhou H. Wang B. Yang Y. Jia Q. Qi Z. Zhang A. (2019). Wxosomes in ischemic heart disease: Novel carriers for bioinformation. Biomed. Pharmacother. 120, 109451. 31586900 10.1016/j.biopha.2019.109451

[B119] Zhu J. Liu B. Wang Z. Wang D. Ni H. Zhang L. (2019). Exosomes from nicotine-stimulated macrophages accelerate atherosclerosis through miR-21-3p/PTEN-mediated VSMC migration and proliferation. Theranostics 9 (23), 6901–6919. 10.7150/thno.37357 31660076 PMC6815950

